# Dimeth­yl(3-oxo-3-phenyl­prop­yl)aza­nium tetra­chloridoferrate(III)

**DOI:** 10.1107/S1600536812018600

**Published:** 2012-04-28

**Authors:** Lei Jin

**Affiliations:** a College of Chemistry and Chemical Engineering, Southeast University, Nanjing 210096, People’s Republic of China

## Abstract

In the title mol­ecular salt, (C_11_H_16_NO)[FeCl_4_], an intra­molecular N—H⋯O hydrogen bond in the cation generates an *S*(6) loop and the conformation of the C(=O)—C—C—N chain is *gauche* [torsion angle = 57.0 (6)°]. The anion is a near-regular tetra­hedron [range of Cl—Fe—Cl angles = 107.93 (8)–112.13 (10)°]. There are no directional inter-ionic bonds in the crystal.

## Related literature

For related structures, see: Hay & Geib (2005 ▶); Ton & Bolte (2004 ▶).
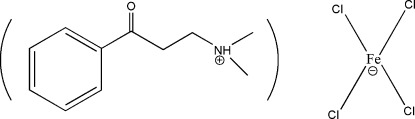


## Experimental

### 

#### Crystal data

(C_11_H_16_NO)[FeCl_4_]
*M* *_r_* = 375.90
Monoclinic, 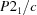

*a* = 6.3166 (13) Å
*b* = 15.149 (3) Å
*c* = 17.293 (4) Å
β = 92.55 (3)°
*V* = 1653.1 (6) Å^3^
*Z* = 4
Mo *K*α radiation
μ = 1.55 mm^−1^
*T* = 291 K
0.26 × 0.22 × 0.20 mm

#### Data collection

Rigaku Mercury2 diffractometer
Absorption correction: multi-scan (*CrystalClear*; Rigaku, 2005 ▶) *T*_min_ = 0.08, *T*_max_ = 0.12
15234 measured reflections
3244 independent reflections
1895 reflections with *I* > 2σ(*I*)
*R*_int_ = 0.080

#### Refinement

*R*[*F* ^2^ > 2σ(*F* ^2^)] = 0.071
*wR*(*F* ^2^) = 0.172
*S* = 1.11
3244 reflections
164 parameters
61 restraints
H-atom parameters constrained
Δρ_max_ = 0.80 e Å^−3^
Δρ_min_ = −0.45 e Å^−3^

### 

Data collection: *CrystalClear* (Rigaku, 2005 ▶); cell refinement: *CrystalClear*; data reduction: *CrystalClear*; program(s) used to solve structure: *SHELXS97* (Sheldrick, 2008 ▶); program(s) used to refine structure: *SHELXL97* (Sheldrick, 2008 ▶); molecular graphics: *SHELXTL* (Sheldrick, 2008 ▶); software used to prepare material for publication: *SHELXTL*.

## Supplementary Material

Crystal structure: contains datablock(s) I, global. DOI: 10.1107/S1600536812018600/hb6733sup1.cif

Structure factors: contains datablock(s) I. DOI: 10.1107/S1600536812018600/hb6733Isup2.hkl

Additional supplementary materials:  crystallographic information; 3D view; checkCIF report

## Figures and Tables

**Table 1:** Selected bond lengths (Å)

Fe1—Cl1	2.164 (2)
Fe1—Cl2	2.1854 (18)
Fe1—Cl3	2.180 (2)
Fe1—Cl4	2.179 (2)

**Table 2:** Hydrogen-bond geometry (Å, °)

*D*—H⋯*A*	*D*—H	H⋯*A*	*D*⋯*A*	*D*—H⋯*A*
N1—H1*D*⋯O1	0.91	2.06	2.735 (7)	130

**Fig. 1.**
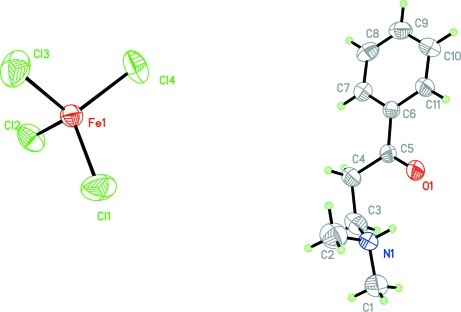
The molecular structure of the title compound, showing 30% probability displacement ellipsoids.

## supplementary crystallographic information

### Experimental

At room temperature, dimethyl-(3-oxo-3-phenyl-propyl)-amine (5 mmol, 0.89 g) was dissolved in 30 ml water, then FeCl_3_.6H_2_O (5 mmol, 1.35 g) was added slowly with sirring. Orange plates were obtained by the slow evaporation of the above filtrate after a week in air.

### Crystal data

**Table:**

(C_11_H_16_NO)[FeCl_4_]	*Z* = 4
*M**_r_* = 375.90	*F*(000) = 764
Monoclinic, *P*2_1_/*c*	*D*_x_ = 1.510 Mg m^−^^3^
Hall symbol: -P 2ybc	Mo *K*α radiation, λ = 0.71073 Å
*a* = 6.3166 (13) Å	θ = 3.2–26°
*b* = 15.149 (3) Å	µ = 1.55 mm^−^^1^
*c* = 17.293 (4) Å	*T* = 291 K
β = 92.55 (3)°	Block, yellow
*V* = 1653.1 (6) Å^3^	0.26 × 0.22 × 0.20 mm

### Data collection

**Table:**

Rigaku Mercury2 diffractometer	3244 independent reflections
Radiation source: fine-focus sealed tube	1895 reflections with *I* > 2σ(*I*)
Graphite monochromator	*R*_int_ = 0.080
Detector resolution: 13.6612 pixels mm^-1^	θ_max_ = 26.0°, θ_min_ = 3.2°
CCD_Profile_fitting scans	*h* = −7→7
Absorption correction: multi-scan (*CrystalClear*; Rigaku, 2005)	*k* = −18→18
*T*_min_ = 0.08, *T*_max_ = 0.12	*l* = −21→21
15234 measured reflections	

### Refinement

**Table:**

Refinement on *F*^2^	Secondary atom site location: difference Fourier map
Least-squares matrix: full	Hydrogen site location: inferred from neighbouring sites
*R*[*F*^2^ > 2σ(*F*^2^)] = 0.071	H-atom parameters constrained
*wR*(*F*^2^) = 0.172	*w* = 1/[σ^2^(*F*_o_^2^) + (0.0538*P*)^2^ + 2.3762*P*] where *P* = (*F*_o_^2^ + 2*F*_c_^2^)/3
*S* = 1.11	(Δ/σ)_max_ < 0.001
3244 reflections	Δρ_max_ = 0.80 e Å^−^^3^
164 parameters	Δρ_min_ = −0.45 e Å^−^^3^
61 restraints	Extinction correction: *SHELXL97* (Sheldrick, 2008), Fc^*^=kFc[1+0.001xFc^2^λ^3^/sin(2θ)]^-1/4^
Primary atom site location: structure-invariant direct methods	Extinction coefficient: 0.0126 (14)

### Special details

**Table:**

Geometry. All e.s.d.'s (except the e.s.d. in the dihedral angle between two l.s. planes) are estimated using the full covariance matrix. The cell e.s.d.'s are taken into account individually in the estimation of e.s.d.'s in distances, angles and torsion angles; correlations between e.s.d.'s in cell parameters are only used when they are defined by crystal symmetry. An approximate (isotropic) treatment of cell e.s.d.'s is used for estimating e.s.d.'s involving l.s. planes.
Refinement. Refinement of *F*^2^ against ALL reflections. The weighted *R*-factor *wR* and goodness of fit *S* are based on *F*^2^, conventional *R*-factors *R* are based on *F*, with *F* set to zero for negative *F*^2^. The threshold expression of *F*^2^ > σ(*F*^2^) is used only for calculating *R*-factors(gt) *etc*. and is not relevant to the choice of reflections for refinement. *R*-factors based on *F*^2^ are statistically about twice as large as those based on *F*, and *R*- factors based on ALL data will be even larger.

### Fractional atomic coordinates and isotropic or equivalent isotropic displacement parameters (Å^2^)

**Table:**

	*x*	*y*	*z*	*U*_iso_*/*U*_eq_	
C1	−0.0240 (17)	0.4076 (6)	0.9427 (6)	0.135 (3)	
H1A	−0.0142	0.3960	0.9974	0.203*	
H1B	−0.1631	0.4292	0.9285	0.203*	
H1C	0.0797	0.4510	0.9301	0.203*	
C2	0.0080 (15)	0.3420 (6)	0.8157 (5)	0.120 (3)	
H2A	0.0347	0.2878	0.7890	0.180*	
H2B	0.1140	0.3847	0.8037	0.180*	
H2C	−0.1294	0.3641	0.7995	0.180*	
C3	−0.1593 (13)	0.2645 (5)	0.9215 (5)	0.102 (2)	
H3A	−0.2943	0.2879	0.9020	0.122*	
H3B	−0.1629	0.2603	0.9774	0.122*	
C4	−0.1252 (11)	0.1715 (4)	0.8870 (4)	0.0829 (19)	
H4A	−0.2390	0.1335	0.9027	0.099*	
H4B	−0.1363	0.1760	0.8310	0.099*	
C5	0.0813 (10)	0.1284 (4)	0.9097 (3)	0.0627 (16)	
C6	0.1018 (9)	0.0308 (4)	0.9021 (3)	0.0547 (14)	
C7	−0.0662 (11)	−0.0227 (4)	0.8783 (4)	0.0745 (19)	
H7A	−0.1985	0.0019	0.8663	0.089*	
C8	−0.0368 (14)	−0.1134 (5)	0.8724 (4)	0.088 (2)	
H8A	−0.1491	−0.1495	0.8559	0.106*	
C9	0.1574 (15)	−0.1496 (5)	0.8908 (4)	0.088 (2)	
H9A	0.1770	−0.2102	0.8863	0.105*	
C10	0.3218 (13)	−0.0976 (5)	0.9157 (5)	0.087 (2)	
H10A	0.4528	−0.1225	0.9292	0.104*	
C11	0.2933 (10)	−0.0084 (4)	0.9206 (4)	0.0701 (18)	
H11A	0.4071	0.0269	0.9371	0.084*	
Cl1	0.4836 (3)	0.50465 (14)	0.21112 (15)	0.1142 (8)	
Cl2	0.0148 (3)	0.38087 (12)	0.17367 (12)	0.0850 (6)	
Cl3	0.4451 (4)	0.3546 (2)	0.05173 (13)	0.1258 (9)	
Cl4	0.4761 (3)	0.26961 (14)	0.24300 (13)	0.0987 (7)	
Fe1	0.36032 (13)	0.37873 (5)	0.17073 (5)	0.0576 (3)	
N1	0.0153 (9)	0.3257 (3)	0.9003 (3)	0.0745 (14)	
H1D	0.1432	0.3026	0.9159	0.089*	
O1	0.2302 (8)	0.1727 (3)	0.9339 (3)	0.0893 (16)	

### Atomic displacement parameters (Å^2^)

**Table:**

	*U*^11^	*U*^22^	*U*^33^	*U*^12^	*U*^13^	*U*^23^
C1	0.165 (7)	0.114 (6)	0.123 (6)	0.047 (6)	−0.041 (6)	−0.034 (5)
C2	0.120 (6)	0.129 (6)	0.113 (6)	0.022 (5)	0.023 (5)	−0.006 (5)
C3	0.100 (5)	0.096 (4)	0.109 (5)	0.026 (4)	0.006 (4)	0.010 (4)
C4	0.080 (4)	0.065 (4)	0.102 (4)	0.010 (3)	−0.014 (3)	0.003 (3)
C5	0.074 (4)	0.057 (4)	0.056 (4)	0.002 (3)	−0.011 (3)	0.005 (3)
C6	0.064 (4)	0.055 (4)	0.045 (3)	0.003 (3)	0.003 (3)	0.002 (3)
C7	0.079 (5)	0.071 (5)	0.072 (5)	0.001 (4)	−0.007 (4)	−0.005 (4)
C8	0.103 (6)	0.069 (5)	0.092 (6)	−0.020 (5)	0.001 (4)	−0.018 (4)
C9	0.122 (7)	0.061 (4)	0.081 (5)	0.010 (5)	0.011 (5)	−0.005 (4)
C10	0.089 (5)	0.071 (5)	0.100 (6)	0.022 (4)	0.001 (4)	−0.002 (4)
C11	0.072 (4)	0.062 (4)	0.076 (5)	0.005 (3)	−0.001 (3)	0.004 (3)
Cl1	0.0904 (15)	0.0800 (13)	0.170 (2)	−0.0095 (11)	−0.0163 (14)	−0.0386 (14)
Cl2	0.0490 (9)	0.0881 (13)	0.1176 (16)	0.0042 (9)	0.0013 (9)	0.0156 (11)
Cl3	0.1048 (16)	0.197 (3)	0.0762 (14)	0.0392 (17)	0.0098 (11)	−0.0246 (15)
Cl4	0.0720 (12)	0.0990 (14)	0.1228 (17)	0.0033 (11)	−0.0227 (11)	0.0345 (12)
Fe1	0.0481 (5)	0.0582 (6)	0.0660 (6)	0.0028 (4)	−0.0023 (4)	−0.0042 (4)
N1	0.081 (3)	0.061 (3)	0.081 (3)	0.013 (3)	−0.011 (3)	−0.004 (3)
O1	0.090 (3)	0.061 (3)	0.113 (4)	0.000 (3)	−0.041 (3)	−0.003 (3)

### Geometric parameters (Å, º)

**Table:**

C1—N1	1.468 (9)	C6—C11	1.372 (8)
C1—H1A	0.9600	C6—C7	1.383 (8)
C1—H1B	0.9600	C7—C8	1.391 (9)
C1—H1C	0.9600	C7—H7A	0.9300
C2—N1	1.483 (10)	C8—C9	1.368 (11)
C2—H2A	0.9600	C8—H8A	0.9300
C2—H2B	0.9600	C9—C10	1.358 (10)
C2—H2C	0.9600	C9—H9A	0.9300
C3—N1	1.499 (9)	C10—C11	1.367 (9)
C3—C4	1.548 (10)	C10—H10A	0.9300
C3—H3A	0.9700	C11—H11A	0.9300
C3—H3B	0.9700	Fe1—Cl1	2.164 (2)
C4—C5	1.495 (8)	Fe1—Cl2	2.1854 (18)
C4—H4A	0.9700	Fe1—Cl3	2.180 (2)
C4—H4B	0.9700	Fe1—Cl4	2.179 (2)
C5—O1	1.214 (7)	N1—H1D	0.9100
C5—C6	1.492 (8)		
			
N1—C1—H1A	109.5	C7—C6—C5	122.6 (6)
N1—C1—H1B	109.5	C6—C7—C8	119.8 (7)
H1A—C1—H1B	109.5	C6—C7—H7A	120.1
N1—C1—H1C	109.5	C8—C7—H7A	120.1
H1A—C1—H1C	109.5	C9—C8—C7	120.0 (7)
H1B—C1—H1C	109.5	C9—C8—H8A	120.0
N1—C2—H2A	109.5	C7—C8—H8A	120.0
N1—C2—H2B	109.5	C10—C9—C8	120.4 (7)
H2A—C2—H2B	109.5	C10—C9—H9A	119.8
N1—C2—H2C	109.5	C8—C9—H9A	119.8
H2A—C2—H2C	109.5	C9—C10—C11	119.5 (7)
H2B—C2—H2C	109.5	C9—C10—H10A	120.2
N1—C3—C4	110.6 (6)	C11—C10—H10A	120.2
N1—C3—H3A	109.5	C6—C11—C10	122.0 (7)
C4—C3—H3A	109.5	C6—C11—H11A	119.0
N1—C3—H3B	109.5	C10—C11—H11A	119.0
C4—C3—H3B	109.5	Cl1—Fe1—Cl4	112.13 (10)
H3A—C3—H3B	108.1	Cl1—Fe1—Cl3	110.63 (11)
C5—C4—C3	115.5 (6)	Cl4—Fe1—Cl3	108.91 (10)
C5—C4—H4A	108.4	Cl1—Fe1—Cl2	108.94 (9)
C3—C4—H4A	108.4	Cl4—Fe1—Cl2	107.93 (8)
C5—C4—H4B	108.4	Cl3—Fe1—Cl2	108.17 (9)
C3—C4—H4B	108.4	C2—N1—C1	110.7 (7)
H4A—C4—H4B	107.5	C2—N1—C3	110.7 (6)
O1—C5—C4	120.2 (6)	C1—N1—C3	105.0 (7)
O1—C5—C6	120.6 (6)	C2—N1—H1D	110.1
C4—C5—C6	119.2 (6)	C1—N1—H1D	110.1
C11—C6—C7	118.3 (6)	C3—N1—H1D	110.1
C11—C6—C5	119.1 (6)		

### Hydrogen-bond geometry (Å, º)

**Table:**

*D*—H···*A*	*D*—H	H···*A*	*D*···*A*	*D*—H···*A*
N1—H1*D*···O1	0.91	2.06	2.735 (7)	130
